# Antimicrobial and Alpha-Amylase Inhibitory Activities of Organic Extracts of Selected Sri Lankan Bryophytes

**DOI:** 10.1155/2020/3479851

**Published:** 2020-08-03

**Authors:** Annalingam Kirisanth, M. N. M. Nafas, Ranga K. Dissanayake, Jayantha Wijayabandara

**Affiliations:** Department of Pharmacy and Pharmaceutical Sciences, Faculty of Allied Health Sciences, University of Sri Jayewardenepura, Nugegoda, Sri Lanka

## Abstract

Medicinal plants have been the main focus of natural product research. However, recent research has revealed that lower plants including bryophytes are also a major resource of biologically active compounds with novel structures. Sri Lanka is considered as a biodiversity hotspot with a higher degree of endemism flora including bryophytes. In this study, different species of bryophytes were investigated for their antimicrobial and alpha-amylase inhibitory activities. The air-dried plant materials of 6 different bryophyte species, *Marchantia* sp., *Fissidens* sp., *Plagiochila* sp., *Sematophyllum demissum*, *Hypnum cupressiforme*, and *Calymperes motley*, were subjected to sequential cold extraction with 3 different organic solvents. All three types of organic crude extracts were subjected to screening of antimicrobial bioassays using the disc-diffusion method against 3 bacterial strains and 1 fungal strain. According to the results obtained, 6 extracts out of 18 showed antibacterial activity for tested Gram-positive bacteria and 1 active against Gram-negative bacteria. Two extracts showed activity against the pathogenic fungus strain. Extracts from some plants were active against tested bacterial as well as fungal species. TLC-based bioautographic study was carried out to identify the corresponding active bands which is useful for active compound isolation. Furthermore, the ethyl acetate extracts were subjected to evaluate alpha-amylase inhibitory activity where three extracts out of six extracts showed moderate inhibitory activity for alpha-amylase with IC50 ranging 8–30%.

## 1. Introduction

The contribution of natural products to the current pharmacopeia is significant while they have provided noticeable leads to novel drug discoveries [1]. The exact number of drugs which are derived from natural products is doubtful. However, reliable estimates reveal that the amount of natural product contribution to the current drug market is not less than 50%. In the case of anticancer and anti-infective agents, the proportion is even higher, and estimate is that almost two-thirds of such agents are derived from natural products [2, 3]. It is notable that less than 10% of the world's biodiversity has been evaluated for potential biological activity, and hence, many more useful natural lead compounds are yet to be discovered [1, 4, 5].

Sri Lanka is considered as a biodiversity hotspot comprised of a rich biological diversity of plant species with a high degree of endemism [6, 7]. The indigenous flora of Sri Lanka comprises about 7,500 plant species. Hot and humid climate with annual rainfall over 2,500 mm makes this island home to different varieties of bryophytes, and most of them are endemic and native [8, 9]. There are over 200 recorded species of bryophytes in Sri Lanka, and most of them are found in tropical rain and submontane and montane forests [10, 11].

Bryophytes are placed taxonomically between algae and pteridophytes and are divided into three classes: mosses, liverworts, and hornworts [12, 13]. Bryophytes are rarely used for herbal medications as medicinal plants. Therefore, the number of research studies carried out based on bryophytes is still low. However, hundreds of novel natural products have been isolated from bryophytes including polysaccharides, lipids, rare amino acids, terpenoids, phenylpropanoids, quinones, and many other specialized metabolites [13–15]. Thus, lower plants including bryophytes of Sri Lanka represent an almost completely uninvestigated, untapped, yet a significant and unique resource for the discovery of new biologically active natural products.

In this study, 6 species of bryophytes from different ecological niches were collected and authenticated. The crude organic extracts, hexane, ethyl acetate, and methanol, were subjected to evaluation for their biological activities such as antimicrobial and alpha-amylase inhibitory activities.

## 2. Materials and Methods

### 2.1. Plant Material Collection and Authentication

Whole plants of 6 plant varieties were collected from Kadugannawa (latitude of 7.255°N 80.5188°E), Kandy district, in the Central Province of Sri Lanka, on October 2019. The morphological and microscopic features of the fresh samples were recorded for authentication purposes.

Due to the lack of herbarium specimens for lower plants, especially bryophytes, the plants were identified and authenticated by scientist Isuru Udayanga Kariyawasam, Molecular Plant Science (Phylogenomics of Bryophytes), at University of Edinburgh College of Science and Engineering, UK.

### 2.2. Preparation of the Extracts

Air-dried and pulverized plant materials were subjected to serial extraction with sonication using hexane, ethyl acetate, and methanol, respectively. The resulting extracts were filtered, and the filtrates were evaporated to dryness under reduced pressure using rotary evaporation (model: RV 10 B, made by IKA, German) at 40°C. The resulted crude extracts were transferred into preweighted glass vials. The dry samples were stored at 4°C until further use.

### 2.3. Chemicals and Reagents

The chemicals and reagents used in this study were hexane, ethyl acetate (EtOAc), methanol (MeOH), 70% ethanol, sodium chloride solution, McFarland standard, 3-(4,5-dimethylthiazol-2-yl)-2,5-diphenyltetrazolium bromide (MTT), dimethyl sulfoxide (DMSO), disodium hydrogen phosphate (Na_2_HPO_4_), sodium dihydrogen phosphate (NaH_2_PO_4_), porcine pancreas alpha-amylase, NaOH, 3,5-dinitrosalicylic acid (DNS), sodium potassium tartrate, and distilled water.

### 2.4. Test Microorganisms

Human pathogenic bacteria *Bacillus subtilis* (UBC 344), *Staphylococcus aureus* (ATCC 25923), and *Pseudomonas aeruginosa* (ATCC 9027) and human pathogenic fungi *Candida albicans* (ATCC 90028) were obtained from the Department of Microbiology, Faculty of Medical Sciences, University of Sri Jayewardenepura and Industrial Technology Institute (ITI), Sri Lanka.

### 2.5. Determination of Antimicrobial Activity

The resulting crude bryophyte extracts were tested, in triplicate, for activity against three pathogenic bacteria, *Bacillus subtilis* (UBC 344), *Staphylococcus aureus* (ATCC 25923), and *Pseudomonas aeruginosa* (ATCC 9027) and one pathogenic fungi, *Candida albicans* (ATCC 90028), at 500 *µ*g/disc concentrations using the standard agar disc-diffusion assay described by the American Clinical Laboratory Standard handbook with few modifications. In brief, cell suspensions of the test microorganisms equal to 0.5 McFarland were prepared using a 24 hrs old culture. A volume of 5 mL of each cell suspension was dispensed onto the surface of dried Mueller-Hinton agar (MHA powder (Hardy, USA), 38.0 g in 1,000 mL of distilled water) dishes, distributed all over the surface, and the excess suspension was removed (positive control: gentamicin (20 *µ*g/disc); negative control: methanol). After overnight incubation, the mean diameters of the inhibition zones were recorded. Samples were dissolved in MeOH to make the final concentration 50 mg mL^−1^ accordingly. Then, 10 *μ*L of the sample was delivered onto the sterile blank disc (WhatmanTM grade AA filter paper discs of 6 mm) to make the final concentration of 500 *μ*g per disc. As the negative control, 10 *μ*L of MeOH was delivered onto a sterile blank disc. Gentamicin (20 *μ*g/disc) was used as the positive control for bacteria, while 1 : 1 mixture of ketoconazole and itraconazole (10 *μ*g/disc from each) was used for pathogenic fungus. The growth inhibitions were visually examined by comparing with the positive control [16–19].

### 2.6. Alpha-Amylase Inhibitory Assay

The EtOAc fraction of crude extracts was evaluated for their alpha-amylase inhibitory activities. The assay was performed according to the published protocol by Wickramarthne et al. with in-house modifications [20]. pH 7.0, 100 mM phosphate buffer solution was used as the reaction medium. The amylase stock solution with a concentration of 2,500 units/mL was prepared by dissolving 25 mg (1,000 unit/mg) of porcine pancreas alpha-amylase in 10 ml of phosphate buffer using the vortex mixer followed by centrifugation at 2,500 rpm. The plant extracts were dissolved in DMSO with the concentration of 5 mg/mL. Then, supernatant was separated and stored in reduced temperature. Initially, 30 *μ*L of the enzyme and 40 *μ*L plant extracts were mixed, and final volume of the reaction mixture was made up to 400 *μ*L using phosphate buffer. Then, the mixture was preincubated for 10 minutes. After 10 minutes, 200 *μ*L 1% of starch was added, and the mixture was incubated at 37°C for 30 minutes. After 30 minutes, the reaction was terminated by addition of 400 *μ*L DNS to the mixture. Then, it was kept in a boiling water bath for 8 minutes and allowed to cool in a water bath. Absorbance was taken at 540 nm (SpectraMax Plus384, Molecular Devices, USA) after proper dilution with distilled water. Control experiments were conducted in an identical way, replacing the extract with 40 *μ*L of DMSO as the negative control and 40 *μ*L acarbose (10 mg/mL) as the positive control. For sample blank incubations (to allow for absorbance produced by the extract), the enzyme solutions were replaced with buffer, and the same procedure was carried out.

The results were expressed as % inhibition which was calculated using the following formula:

(1) $$\begin{array}{c} \text{inhibition activity }\left(\text{\%}\right)=\frac{\text{absorbance }\left(\text{control}\right)-\text{absorbance }\left(\text{test}\right)}{\text{absorbance }\left(\text{control}\right)}\times 100, \end{array}$$

(2) $$\begin{array}{c} \text{inhibition compared to acarbose}=\frac{\text{\% inhibition of test}}{\text{\% inhibition of positive control}}\times 100, \end{array}$$

### 2.7. Bioautographic Analysis

The extracts which showed promising antimicrobial activities were subjected to analysis using bioautographic techniques in order to identify respective antimicrobial active bands. *S*. *aureus* was used as the testing microbe since most of the extracts showed highest activity against *S*. *aureus*. As an exception, in this assay, the cell suspension was diluted to make the final OD (0.0001) by using melted MHA in 0.6%. The TLC plate was then overlaid with the diluted cell suspension of the test microorganism suspended in 0.6% MHA. Agar was allowed to solidify at room temperature and incubated at 37°C for 16–24 h. After the incubation period, in order to identify active bands, the TLC plate with MHA was flooded with a solution of MTT, 2 mg mL^−1^ (in sterile distilled water), and was incubated for 1 hr at 37°C.

## 3. Results

**Figure 1:**
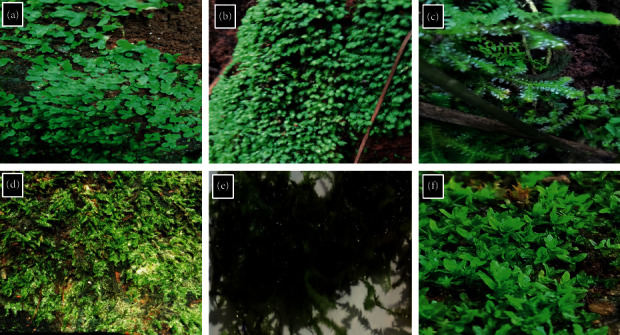
*In situ* pictures of the collected bryophytes: (a) *Marchantia* sp., (b) *Fissidens* sp., (c) *Plagiochila* sp., (d) *Sematophyllum demissum*, (e) *Hypnum cupressiforme*, and (f) *Calymperes motley*.

Less vulnerable bryophyte species were collected based on their availability. The collected bryophytes were identified based on macroscopic and microscopic morphological features. According to the features, the bryophyte species were authenticated as *Marchantia* sp. (MR), *Fissidens* sp. (FS), *Plagiochila* sp. (PG), *Sematophyllum demissum* (SD), *Hypnum cupressiforme* (HC), and *Calymperes motley* (CM). The morphology of the above bryophytes is given in Figure 1.

**Figure 2:**
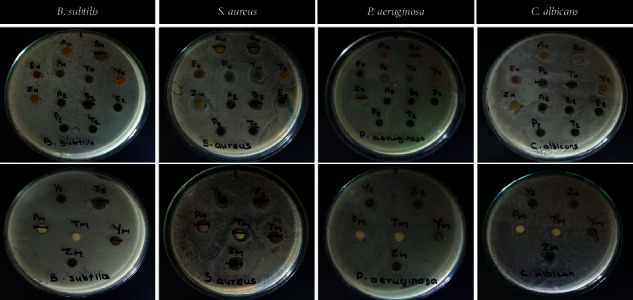
Antimicrobial activities of crude organic extracts against *B. subtilis*, *S. aureus*, *P. aeruginosa*, and *C. albicans* at 500 *µ*g/disc concentration using the disc-diffusion method.

In total, 18 extracts (hexane, EtOAc, and MeOH extracts of six bryophytes) were screened for their antimicrobial potential. The antimicrobial activities of crude extracts are given in Figure 2, and positive results are summarized in Table 1. Out of the 18 extracts, 10 extracts did not show any activity against the tested microorganisms. Most promising activities were shown against tested Gram-positive bacteria. Only the hexane extract of *Fissidens* sp. (FS-Hex) showed the activity against *P*. *aeruginosa*. Only hexane extracts of *S*. *demissum* (SD-Hex) and *H*. *cupressiforme* (HC-Hex) showed antifungal activity. None of the *Plagiochila* sp. extract showed any antimicrobial activity.

**Figure 3:**
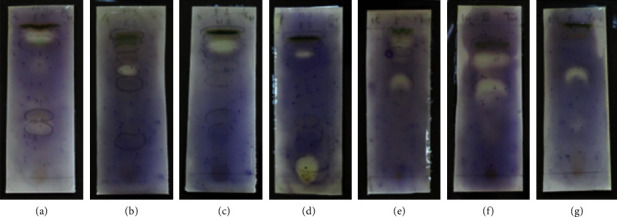
Bioautographic analysis of antimicrobial active crude extracts of (a) FS-Hex, (b) SD-Hex, (c) HC-Hex, (d) FS-EtOAc, (e) SD-MeOH, (f) HC-MeOH, (g) and CM-MeOH against *S*. *aureus*.

The bioautographic analysis of the antimicrobial active extracts against *S*. *aureus* is given in Figure 3. The clear zone indicates the respective TLC band which has antimicrobial potential. All extracts showed a simple bioautographic pattern with less than 3 active bands. Most of active compounds are between nonpolar to medium polar. Therefore, the respective active compounds can be easily separated via normal phase silica column chromatography since the polar normal phase has more selectivity towards nonpolar and medium polar compounds.

The alpha-amylase inhibitory activities of the EtOAc extract are given in Table 2. According to the results, some extracts showed moderate inhibitory potential. EtOAc extract of *Fissidens* sp. showed the highest inhibitory activity (39%) followed by *Marchantia* sp. (23%). Two extracts (*S*. *demissum* and *C*. *motley*) were completely inactive.

## 4. Discussion

Only a handful of studies have been carried out to investigate the bioactive potential of lower plants including bryophytes [13, 21, 22]. Since Sri Lanka is an isolated island with remarkable biodiversity among its flora, the density and the number of bryophyte species are very high. In this study, commonly available six bryophyte species were collected and authenticated as *Marchantia* sp., *Fissidens* sp., *Plagiochila* sp., *S*. *demissum*, *H*. *cupressiforme*, and *C*. *motley*. There are two liverworts (*Marchantia* sp. and *Plagiochila* sp.), and the rest of bryophytes are mosses.

*Marchantia* sp. are a good source of bioactive metabolites, and a number of compounds were isolated from different *Marchantia* sp. [23, 24]. Marchantiaceae is known to contain a large amount of marchantin-type cyclic bisbibenzyls, which contain different types of biological activities, including cytotoxic, antimicrobial, anticancer, calmodulin inhibitory, and cardiotonic activities [25–27]. *Fissidens* sp. and *Plagiochila* sp. are also good sources of secondary metabolites. *Plagiochila barteri* and *Plagiochila terebrans* are known to produce cyclic bisbibenzyls, ent-spathulenol, 1(10),14-halimadien-13*ξ*-ol, trifarienol B (11), and marchantins C and H, with antimicrobial, anticancer, and antiviral properties [28–30]. However, there are no scientific evidences for isolation and biological screening of bioactive metabolites from *S*. *demissum* and *C*. *motley*. Therefore, this is the first record to investigate bioactive potential of these bryophytes.

According to the disc-diffusion and bioautographic data of this study, most of the active compounds are in the nonpolar region. This is because bryophytes are lower plants, and they do not have special mechanism to preserve water. Hence, they produce different types of oil- and fat-based compounds in order to avoid loss of water, and most of the secondary metabolites of lower plants are lipophilic. The bioautographic analysis revealed the distribution of antimicrobial compounds. Normally, the bioautogram of higher plants is very complex and consists of several active compounds. However, the number of active compounds of the bryophytes used this study is limited to maximum 3. Therefore, isolation of antimicrobial compounds from these plants is quite easy compared to the higher plants. Furthermore, bioautography is a very valuable and time-saving method for bioassay-guided isolation of antimicrobial active compounds.

Except 2 extracts, the other tested EtOAc extracts did not show potent inhibitory activity for alpha-amylase. The most promising inhibitory activity was shown by the EtOAc extract of *Fissidens* sp.; therefore, this is a valuable finding, and the extract can be used to isolate respective active metabolites since isolation of antialpha-amylase compounds from lower plants is very rare. Furthermore, alpha-amylase inhibitory activity represents *in vitro* antidiabetic activity. Hence, these active extracts are useful to regulate blood glucose levels and act as postprandial glucose regulators. There are only few studies on alpha-amylase inhibitory activity of bryophytes, and this study is one of them.

Apart from the valuable finding of this study in the methodology section, all procedures are given in an explanative manner because most of the published articles mentioned these protocols briefly, and researchers are encountering problem while repeating these experiments. Therefore, researchers who are carrying out antimicrobial, bioautography, and alpha-amylase inhibitory assays can easily follow the given procedure.

## 5. Conclusion

The results of this study show that the Sri Lankan bryophytes are capable of producing antimicrobial metabolites, active against both Gram-negative and Gram-positive bacteria, and antialpha-amylase activity and thus are potential sources for the discovery of new bioactive substances that may prove to be clinically useful. This is the first record of investigating bioactive potential of Sri Lankan bryophytes. Therefore, further studies on lower plants including bryophytes bring out their hidden biosynthetic capabilities, along with wider bioactivity testing which can be expected to immensely enhance the value of this valuable and underutilized resource.

## Figures and Tables

**Table 1:** Antimicrobial activity of the crude extracts at 500 *µ*g/disc concentrations.

Plant extracts	Antimicrobial activity			
	Mean diameter of the inhibition zone (mm) ± SE (500 mg/disc)			
	*S*. *aureus*	*B*. *subtilis*	*P*. *aeruginosa*	*C*. *albicans*
MR-Hex	8.3 ± 0.2	—	—	—
FS-Hex	10.5 ± 0.3	7.6 ± 0.2	7.3 ± 0.2	—
FS-EtOAc	8.3 ± 0.2	9.2 ± 0.1	—	—
SD-Hex	—	—	—	7.4 ± 0.2
SD-MeOH	12.3 ± 0.2	7.4 ± 0.1	—	—
HC-Hex	—	—	—	7.8 ± 0.2
HC-MeOH	8.5 ± 0.2	—	—	—
CM-MeOH	8.3 ± 0.2	7.3 ± 0.2	—	—
+ve control	23.5 ± 0.4	20.3 ± 0.2	15.2 ± 0.1	13.1 ± 0.8
−ve control	—	—	—	—

Hex: hexane extract, EtOAc: ethyl acetate extract, and MeOH: methanol extract.

**Table 2:** Results of alpha-amylase inhibitory activities of the EtOAc extract.

Plant extract	Inhibition activity percentage (1)	Inhibition compared to acarbose (2) (%)
*Marchantia* sp.	23 ± 3	35 ± 2
*Fissidens* sp.	39 ± 2	59 ± 4
*Plagiochila* sp.	12 ± 1	18 ± 3
*S*. *demissum*	Inactive	—
*H*. *cupressiforme*	8 ± 2	12 ± 3
*C*. *motley*	Inactive	—
Positive	66 ± 5	100

## Data Availability

The data used to support the findings of this study are available from the corresponding author upon request.
